# Reconfirming the Traditional Model of HIV Particle Assembly

**DOI:** 10.1371/journal.pbio.0040445

**Published:** 2006-12-05

**Authors:** Liza Gross

For HIV infection to take hold, a virus particle must first attach to a cell to gain entry. Once inside, the viral genome is reverse transcribed from RNA to DNA, and then integrated into the host genome. By co-opting the host’s molecular machinery, the virus churns out multiple copies of its genome (transcribed back into RNA), and viral components are synthesized and assembled into particles that leave the cell and begin the cycle anew. Viral assembly is mediated by the virus’s major structural protein, Gag, which can be fused to another viral protein, Pol, which encodes the virus’s enzymes.

In the traditional model, Gag proteins assemble at the plasma membrane prior to release. Multiple studies have recently challenged this model, however, showing abundant Gag proteins and mature virions in structures called endosomes, which take up extracellular molecules and particles through a process called endocytosis. These studies proposed that Gag is first sent to endosomal membranes before reaching the plasma membrane or extracellular space through an endosome-based transport pathway.

Initial studies described this endosomal assembly pathway as unique to macrophages, the primary targets of HIV infection, though other studies have suggested it may occur in all cell types. In a new study, Nolwenn Jouvenet, Paul Bieniasz, and their colleagues combined pharmacological, genetic, biochemical, and microscopic approaches to determine where HIV assembles in the cell. Their results undercut several elements of the endosomal model and place the traditional model back on a solid foundation.

To track HIV Gag localization and assembly, the authors started by transiently inducing Gag protein expression in a standard experimental cell line (293T cells). About 10 hours after transfecting cells with the *Gag* gene, they detected Gag-Pol processing, an indicator of assembly, and particle release. If Gag targeting to endosomes initiates particle assembly or transport to the membrane, the authors reasoned, then both processes should depend on endosome motility. But when cells were treated with a drug that blocks endosome movement, HIV assembly was unaffected, based on Gag processing and particle release. Further, microscopic analysis showed high levels of fluorescently tagged Gag proteins at the plasma membrane. Thus, they concluded, neither Gag transport to the plasma membrane nor virion assembly relied on endosome motility.

Fluorescently tagged Gag proteins were seen either scattered throughout the cytoplasm, assembled at the plasma membrane (about 4 hours after transfection), or accumulating both in internal compartments and at the plasma membrane (8–10 hours after transfection). The internal compartments turned out to be endosomes (based on characteristic protein markers), and the authors suspected that their accumulated Gag contents had been acquired through endocytosis; when they used genetic tools to block endocytosis, the endosomal Gag accumulations were no longer evident. What’s more, inhibiting endosomal Gag had no effect on virion assembly and release.

**Figure:**
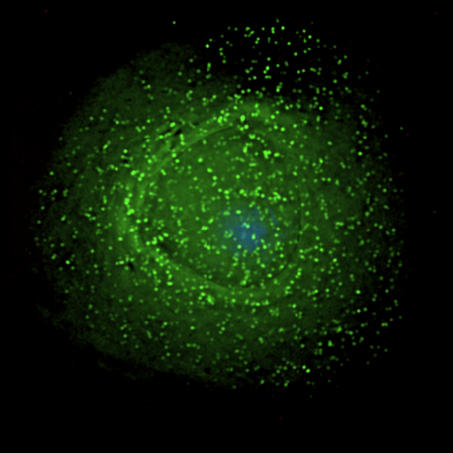
HIV-1 particles assembling at the surface of an infected macrophage.

The authors also manipulated Gag’s membrane-binding domain to assess the consequences on viral assembly and release. If Gag is initially sent to endosomes to trigger HIV assembly and release, then exchanging Gag’s membrane-binding protein with a domain that targets endosomes should precipitate particle assembly and release. But that’s not what the authors found. Gag and Pol proteins that were artificially targeted to the plasma membrane triggered virion assembly and release just as efficiently as their wild-type (nonmanipulated) counterparts, but direct targeting of Gag proteins to endosomes resulted in particle assembly in endosomes but little or no particle release.

The authors repeated these experiments in macrophages, one of the virus’s natural targets, using a new technique that facilitates transfection in macrophages. Localization patterns of newly synthesized Gag proteins followed a temporal pattern similar to that seen in the 293T cells. Just 4–6 hours after transfection, fluorescently tagged Gag proteins were either distributed throughout the cytoplasm or clustered at the plasma membrane. At 24 hours, Gag was also seen in internal compartments. And similar to what was found in 293T cells, inhibiting endosomal transport in macrophages revealed that endosomes cannot support virion assembly and release in these cells either.

Altogether, these results reaffirm the long-established model that Gag-mediated particle assembly occurs at the plasma membrane. Virions seen in endosomes arrive there through an endocytic pathway at a later point, which does not support HIV release. In fact, the authors argue, assuming a slow rate of Gag synthesis and a high rate of plasma membrane internalization, one would expect most HIV Gag particles to wind up in endosomes. Future studies can further explore the kinetics that determine the rate at which virions are released or sequestered in cellular compartments. But the authors argue that their results unequivocally demonstrate that HIV assembly occurs at the plasma membrane.

